# Quality of Life and Working Conditions of Plastic Surgeons and Trainees: A National Survey

**DOI:** 10.3390/ijerph22050778

**Published:** 2025-05-14

**Authors:** Léna G. Dietrich, Michael J. Deml, Laura De Pellegrin, Cédric Zubler

**Affiliations:** 1 Department of Plastic and Hand Surgery, Inselspital University Hospital Bern, University of Bern, 3010 Bern, Switzerland; laura.depellegrin@insel.ch (L.D.P.); cedric.zubler@unibe.ch (C.Z.); 2 Institute of Sociological Research, University of Geneva, 1211 Geneva, Switzerland

**Keywords:** quality of life, working conditions, plastic surgery, job satisfaction

## Abstract

Background: While the well-being and working conditions of healthcare professionals are increasingly scrutinized, there remains a critical research gap regarding the quality of life and job satisfaction of plastic surgeons in Switzerland. No prior national study has systematically examined these aspects within this specialty. Objective: This study aims to address this gap by evaluating workload, career satisfaction, and quality of life among Swiss plastic surgeons and trainees, thereby providing evidence to inform systemic improvements in the profession. Methods: A national, multilingual online survey was distributed to all members of the Swiss Society for Plastic Surgery and the Association of Young Plastic Surgeons. A total of 102 plastic surgeons responded (response rate: 22.7%). The survey assessed contractual versus actual working hours, work performed during personal time, mental health indicators (e.g., burnout), and career satisfaction. Descriptive and correlational analyses were conducted. Results: The respondents reported an average of 58 actual versus 49 contractual working hours per week, with an additional 8.1 h spent working during leisure time. Burnout symptoms were present in 29%, and 63% experienced work-related stress during their free time. While 42% wished to reduce their workload, 88.7% would still choose the profession again. Career satisfaction averaged 3.66/5, although 35% rated their salary as inadequate. Notably, 79.8% reported work negatively affecting private relationships, despite 82.65% feeling supported by their environment. Conclusion: This first nationwide assessment highlights the high workload and psychological strain faced by Swiss plastic surgeons. Key priorities include targeted burnout prevention, structural workload reduction, enhanced support for work–life integration (especially among women and younger surgeons), and improved compensation. These measures are essential to sustain the well-being of practitioners and ensure long-term quality in surgical care.

## 1. Introduction

Patient quality of life is undeniably important and represents a cornerstone of healthcare. However, this study shifts the focus to surgeons themselves, a group that might be often overlooked. Few studies have explored the quality of life, job satisfaction, and burnout rates among surgeons in general, and even fewer have addressed these is-sues specifically within the field of plastic surgery [1,2,3,4]. While most of the existing re-search originates from the United States, studies examining Swiss surgeons, such as hand surgeons, remain limited in scope [1]. The working conditions of Swiss plastic surgeons have come under increasing scrutiny due to rising patient volumes, growing workloads, and the demands of digitalization [5,6]. Simultaneously, heightened media attention on the working conditions of medical residents has illuminated the pressures faced by healthcare professionals [7,8,9]. These developments make it timely and necessary to assess the situation of plastic surgeons in Switzerland, particularly within the context of evolving societal and professional challenges. 

Plastic surgery is a demanding yet rewarding profession requiring technical expertise, mental resilience, and physical endurance. Studies have documented high rates of burnout among surgeons, largely driven by long working hours, bureaucratic pressures, and the emotional toll of patient care. For instance, Prendergast et al. reported that over 40% of plastic surgeons experience burnout, characterized by emotional exhaustion and reduced personal accomplishment, factors that negatively affect both surgeons and patient outcomes [10]. The physical toll of the profession also warrants attention. Nagarkar et al. highlighted the high prevalence of musculoskeletal afflictions among plastic surgeons, often caused by prolonged surgeries and poor ergonomics [11]. Additionally, the physiological stress associated with surgery has been linked to cardiovascular strain, as demonstrated by Demirtas et al., who identified significant changes in surgeons’ cardiac activity during operations [12]. Building on this, a comparative study by Bernburg et al. examined occupational stress, depressive symptoms, and work ability among medical residents across various specialties [13]. The study found that surgeons faced the highest occupational distress but maintained the greatest work ability and lowest depressive symptoms. In contrast, anesthesiologists had the most depressive symptoms, while pediatricians experienced the highest emotional strain, underscoring the need for tailored mental health support.

Mentorship, work–life balance, and gender equity play pivotal roles in shaping the experiences of plastic surgeons. Ibrahim et al. emphasized that millennial medical students prioritize career paths that support personal well-being, with female students particularly concerned about gender equity [14]. These considerations are critical as the field evolves to meet the expectations of a new generation of surgeons. Systemic challenges further compound these individual struggles. Sun et al. described the undervaluation of educational efforts within academic settings, which undermines surgeon satisfaction and job retention [15]. Other challenges are financial pressures, reimbursement issues, or gen-der disparities in leadership and pay [16,17]. Addressing these systemic issues is, therefore, essential to ensure the sustainability of the profession and the well-being of its practitioners.

Insights from other specialties further emphasize the need for intervention. For ex-ample, a survey conducted among gynecologists in France revealed significant gender disparities in how workload affects time for personal life, family, and friends. The study also noted a neglect of physical exercise and mental health among physicians, highlighting the psychological toll of the profession and the urgency for targeted support measures [3]. 

Given these considerations, this study aimed to assess the quality of life and working conditions of Swiss plastic surgeons and trainees. By contextualizing the findings within the broader international literature, this study seeks to identify key areas for intervention.

## 2. Materials and Methods

### 2.1. Survey Development

To evaluate the quality of life and working conditions of plastic surgeons in Switzerland, we developed an online survey tailored specifically to this group. The survey was based on a previously developed template used in a national study on hand surgeons, which was adapted to the specific context of plastic surgery. This earlier instrument was created in collaboration with our co-author (MJD) from the Institute of Sociological Research at the University of Geneva and has been published in *Medicina* [1]. The survey topics and questions were further refined through discussions within a multidisciplinary team that included experts in plastic surgery and sociology. This collaborative approach ensured that the survey was concise, relevant, and addressed the challenges faced by plastic surgeons.

An online survey was developed to gather comprehensive data, building on established methodologies from the literature [18]. The survey was administered using LimeSurvey (LimeSurvey GmbH, 2003, GPL, JavaScript, PHP) and was initially prepared in English. To account for Switzerland’s linguistic diversity, it was professionally translated into German and French by bilingual members of the research team. The respondents were able to select their preferred language at the beginning, ensuring accessibility and ease of participation for surgeons across the country.

To validate the survey, a pilot test was conducted in three languages (English, German, and French) with participants from the target demographic. Their feedback was used to refine the wording and improve the clarity and relevance of the questions. Based on the pilot phase, minor adjustments were made before the survey was distributed. For consistency and accessibility, all study results are presented in English.

### 2.2. Survey Content

The survey comprised 36 questions designed to explore key topics such as working conditions, as well as aspects of private life among Swiss plastic surgeons and trainees. The questions addressed critical aspects of quality of life, including satisfaction with the following areas: profession/career, family life, social life, free time, overall satisfaction, and demographic and background data.

The survey examined variations across demographics (e.g., gender, age, professional position, and workplace type) and their associations with indicators such as chronic fatigue, burnout, or depression. Specific attention was given to exploring links between these factors and the participants’ perceived quality of life. A 5-point Likert scale was used to assess satisfaction in various domains, with options ranging from 1 (“not satisfied at all”) to 5 (“completely satisfied”) for categories such as wages, family life, social life, and career. Overall quality of life was similarly assessed using a scale from 1 (“insufficient”) to 5 (“excellent”). The participants were also asked to reflect on their career choices to determine if they would choose to study medicine again and if they would select plastic surgery as a profession again. As part of the survey, the participants were asked to indicate their current workplace, using the classification system established by the Swiss Institute for Postgraduate and Continuing Medical Education (SIWF/ISFM). In Switzerland, the classification of hospitals into Categories A, B, and C reflects their accreditation status and capacity to provide residency training in accordance with the standards of the Swiss Institute for Postgraduate and Continuing Medical Education (SIWF/ISFM). Category A (Level I) institutions are university-affiliated or large tertiary referral centers that are fully accredited to offer the complete postgraduate curriculum in plastic surgery. They are characterized by high patient volumes, broad subspecialty coverage, and structured teaching programs. Category B (Level II) hospitals provide partial residency training and typically serve as regional centers offering a wide range of services, although with less subspecialization and academic activity compared to Category A. Category C (Level III) institutions are smaller clinics or hospitals with limited training authorizations, generally focused on specific aspects of the specialty [19].

### 2.3. Survey Administration

The survey was available online from 27 September 2023, to 28 April 2024, with most participants completing it within 8 to 12 min. Invitations to participate were extended to all residents and senior physicians listed in the mailing lists of the Swiss Society for Plastic Surgery and the Association of Young Plastic Surgeons (in total, approximately 450 society members). While membership in these societies is not mandatory in Switzerland, it was assumed that the majority of Swiss plastic surgeons and trainees are affiliated with at least one of these organizations. To maximize participation, the societies supported the survey distribution by inviting their members via email, providing details about the sur-vey and its objectives, and sending a follow-up reminder one to two weeks after the initial invitation to enhance response rates. Participation in the survey was entirely voluntary, and anonymity was rigorously maintained throughout the process to ensure that respondents felt secure in providing honest and candid responses. It was possible to skip any questions that the respondents did not wish to answer.

### 2.4. Statistical Analysis

The collected data were analyzed using both univariable and multivariable models to identify patterns and associations across various dimensions of quality of life and professional experiences. Statistical analysis was conducted using the IBM SPSS software package (Version 28, IBM, SPSS, Armonk, NY, USA). All data were presented as percentage rates. To evaluate associations between pairs of categorical variables, Spearman’s correlation and chi-square tests were used as appropriate.

## 3. Results

A total of 102 participants returned the survey. The overall response rate was 22.7%, with 102 respondents out of 450 society members. The demographic details of the participants are summarized in Table 1.

**Figure 1:**
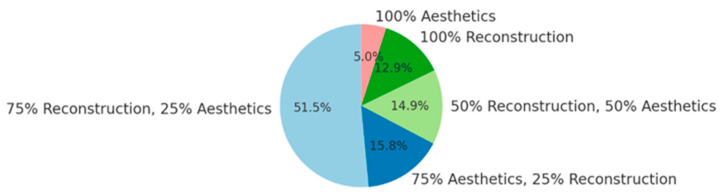
Distribution of professional activities.

The respondents provided answers in all three languages: German (*n* = 87, 85.3%), French (*n* = 3, 2.9%), and English (*n* = 12, 11.8%). Most participants were in the 30–44-year age range (*n* = 39, 38.2%). There were more male participants (*n* = 60, 58.8%) than female (*n* = 42, 41.2%). A detailed breakdown of the gender distribution across professional roles is presented in Figure 1.

Males were more often in leadership roles, with 12 working in leading or chief positions compared to 5 women in such roles. Among female plastic surgeons, 19 were residents, accounting for 45.2% of all female participants. Most respondents were in a relationship (*n* = 76, 74.5%), and 45.1% had children: 16 had one child, 20 had two children, with only a small number (*n* = 10) reporting three or more children.

### 3.1. Professional Details and Working Hours

**Figure 2:**
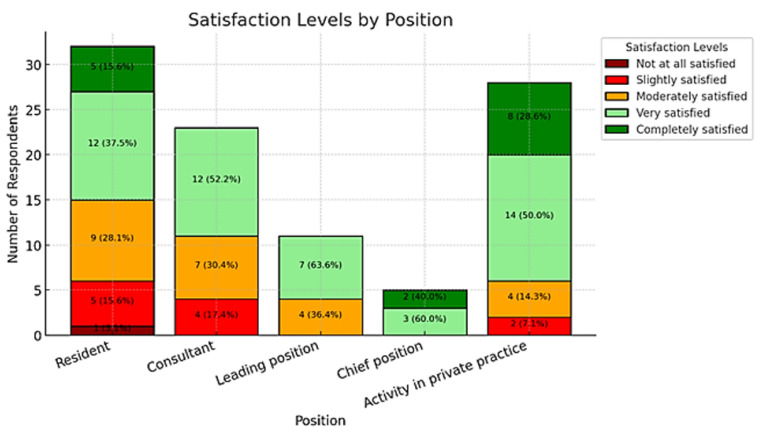
Distribution of quality of life by position.

In total, 47 participants (46.1%) were employed at a Category A clinic (level I), 16 (15.7%) at a Category B clinic (level II), and 5 (4.9%) at a Category C clinic (level III), while 30 (29.4%) worked in private practice. The distribution of professional activities among survey respondents is depicted in Figure 2. 

Regarding their positions, there were 32 residents (31.4%), 23 consultants (22.5%), 11 physicians in leading positions (10.8%), and 28 practice owners/employees (27.5%). The work-related characteristics of the participants are detailed in Table 2. 

Most participants were on call at least 4–7 days per month (*n* = 38, 37.3%), while only 17 (16.7%) reported that they were never on call. The actual average working hours per week varied from the contractual working hours, ranging between 30 and 82 h (mean 58 h). Vacation days ranged from 8 to 60 per year (mean 32 days), and working hours during free time varied from 0 to 30 h (mean 8.1 days). Among all respondents, 65 (46.1%) expressed a desire to work in a leading position, while 60 (53.3%) believed that greater satisfaction could be achieved through more autonomy.

### 3.2. Health Status

**Figure 3:**
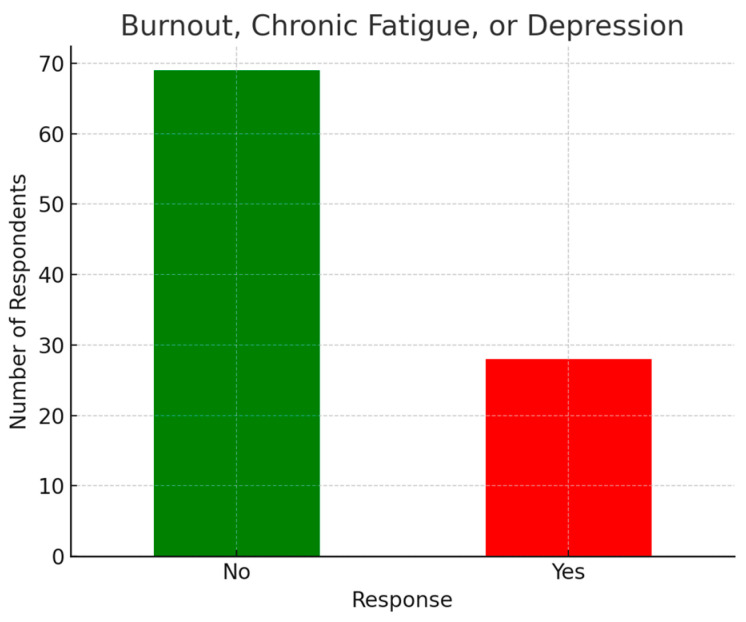
Prevalence of burnout, chronic fatigue, and depression.

Among the respondents, 40 (37.7%) reported an average of 7 h of sleep per night. Additionally, 29 (29%) indicated that they had experienced or were currently suffering from burnout, chronic fatigue, or depression. The prevalence of burnout, chronic fatigue, and depression among respondents is illustrated in Figure 3. 

There were 10 participants (9.4%) who identified as smokers, and 3 (2.8%) reported using micro-stimulants. More than half of the respondents (*n* = 63, 63%) felt stressed or affected by work during their holidays or free time. Specifically, 24 residents (77.4%), 7 consultants (26.1%), 9 leading positions (81.8%), and 12 chief positions or practice owners/employees (42.9%) reported feeling job-related stress during these times. Stress was reported by 31 female respondents (73.81%) and 32 male respondents (55.17%) (*p* = 0.09). 

A total of 99 respondents (93.4%) felt understood and supported by their families, friends, or workplaces regarding their professional challenges. Among the participants, 12 residents (37.5%), 9 consultants (39.1%), 6 leading positions (54.5%), and 2 chief positions or practice owners (6.3%) reported experiencing chronic exhaustion, burnout, or depression. Gender analysis revealed that 16 female plastic surgeons (38.1%) and 13 male plastic surgeons (22.4%) faced issues with depression, burnout, or chronic exhaustion, indicating that women and residents were more likely to suffer from these conditions (gender: *p* = 0.138; position: *p* = 0.0068).

### 3.3. Quality of Life

The satisfaction ratings across various aspects are summarized in Table 3. 

**Figure 4:**
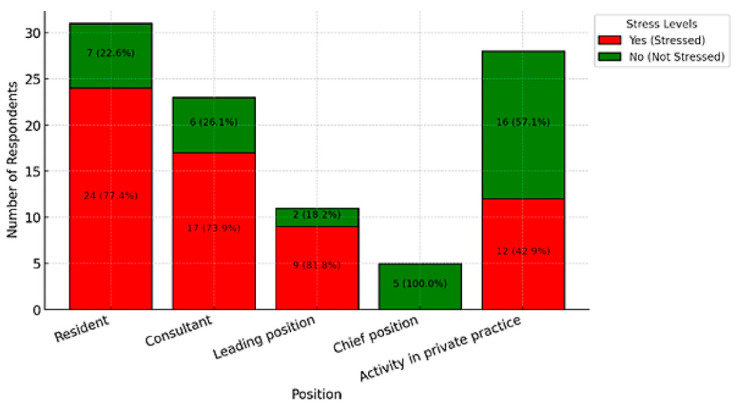
Stress during holidays or leisure time by position.

A total of 42 respondents (39.6%) rated their social life satisfaction as moderate, while 16 (15.1%) considered it insufficient, and 6 (5.7%) stated they had no social life at all. The prioritization of different life areas among respondents is summarized in Figure 4.

Regarding family satisfaction, 43 participants (40.6%) rated it as good, whereas 29 (27.4%) found it moderate, 14 (13.2%) as insufficient, and 12 (11.3%) as unsatisfied. Most respondents evaluated their job satisfaction as good (*n* = 48, 45.3%), while 25 (23.6%) rated it moderate, 11 (10.4%) as insufficient, and 1 (0.9%) as not satisfied at all. Regarding salary satisfaction, 27 respondents (25.5%) rated it moderate, 27 (25.5%) considered it insufficient, and 8 (7.5%) reported being not satisfied at all. Overall, the majority of respondents indicated that work negatively affected their personal relationships, with no precise percentage available in the dataset. In terms of career choices, 64.2% of respondents stated they would choose to study medicine again, while 88.7% confirmed they would pursue plastic surgery as their specialty again. Among different professional positions, five residents (15.2%), nine consultants (39.1%), and six in leadership positions or private practice (18.8%) reported a moderate or poor quality of life, whereas nine (81.8%) physicians in leadership roles expressed overall satisfaction. Furthermore, 42 respondents (39.6%) admitted to having seriously considered leaving the profession.

### 3.4. Correlations

#### 3.4.1. Stress During Holidays or Free Time

**Figure 5:**
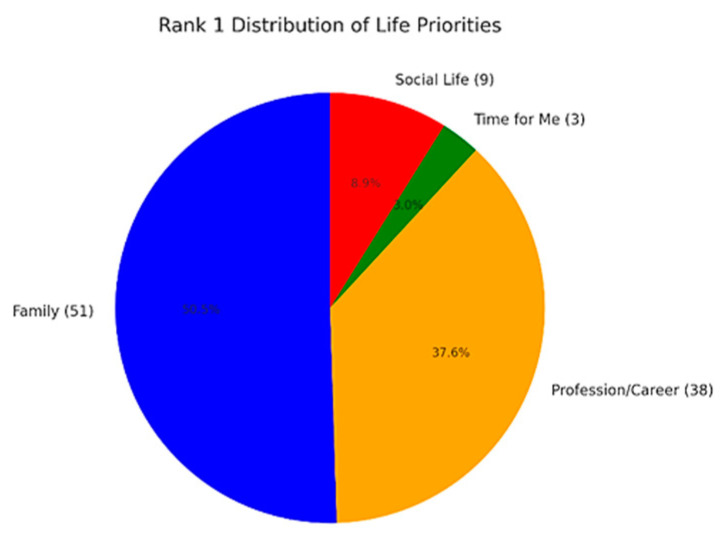
Illustration of life priorities: The figure highlights the item ranked as the number one priority.

The analysis revealed a moderate positive correlation (r = 0.29) between stress during holidays or free time and the likelihood of experiencing burnout, chronic fatigue, or depression. This suggests that individuals who report feeling stressed by their work even during non-working hours are more prone to these mental health challenges. Furthermore, a strong negative correlation (r = −0.53) was identified between stress during holidays and overall quality of life, indicating that an inability to disconnect from work significantly impairs well-being. Among the participants, 24 residents (77.4%), 17 consultants (73.9%), 9 physicians in leadership positions (81.8%), and 12 individuals in private practice (42.9%) reported experiencing work-related stress during their holidays and leisure time. Figure 5 provides a graphical representation of stress levels during holidays or leisure time, stratified by professional position. 

#### 3.4.2. Areas of Life Ranked as Personal Priority

None of the respondents in chief positions reported experiencing stress during these periods. Further analysis revealed a statistically significant association between professional position and the experience of stress during holidays (χ^2^ = 19.05, *p* = 0.0008). Work-related stress during non-working hours is not evenly distributed across roles. It is predominantly prevalent among residents and physicians in leadership positions, whereas individuals in private practice and those in chief roles report significantly lower levels of stress outside of working hours. Additionally, the analysis identified a statistically significant relationship between experiencing stress during holidays or free time and suffering from chronic exhaustion, burnout, or depression (χ^2^ = 6.95, *p* = 0.0084).

### 3.5. Quality of Life

**Figure 6:**
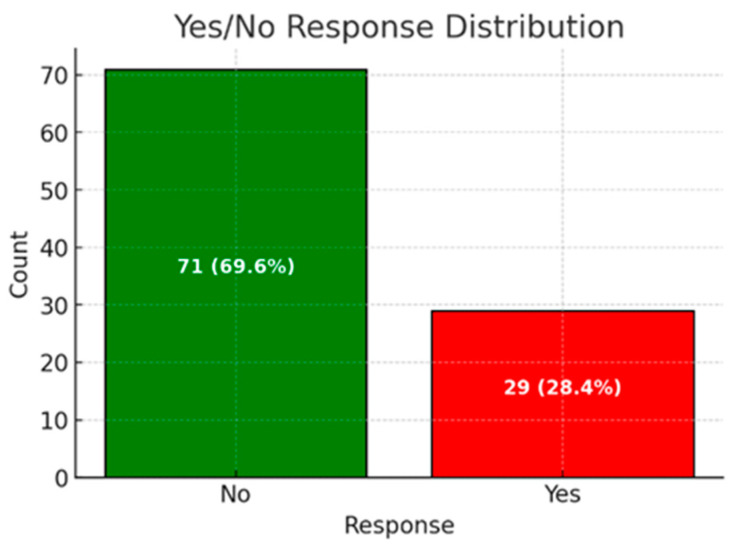
Distribution of chronic exhaustion, burnout, or depression.

The analysis did not reveal significant differences in quality-of-life levels across professional positions. Participants in private practice reported high satisfaction, with 50.0% rating their quality of life as “very good” and 28.6% as “excellent”. Chief physicians demonstrated even greater satisfaction, with 60.0% rating their quality of life as “very good” and 40.0% as “excellent”. Consultants reported a more varied distribution, with 52.2% rating their quality of life as “very good,” while 30.4% rated it as “good.” In contrast, residents experienced lower levels of satisfaction, with 37.5% rating their quality of life as “good”, 28.1% as “very good”, and 15.6% as “moderate”. The variation in quality of life across different professional positions is depicted in Figure 6.

However, a chi-square test showed no statistically significant relationship between professional position and quality-of-life levels (χ^2^ = 21.31, *p* = 0.167), indicating that variations in satisfaction across positions may not be meaningful. Regarding gender, the analysis also showed no significant differences in quality of life. Male participants reported a balanced distribution, with 50.8% rating their quality of life as “very good,” 16.9% as “excellent”, and 23.7% as “good”. Female participants showed a slightly lower satisfaction, with 42.9% rating their quality of life as “very good” and 26.2% as “good”. A chi-square test for gender and quality of life confirmed no statistically significant relationship (χ^2^ = 2.67, *p* = 0.615), suggesting that gender does not have a meaningful impact on quality of life in this dataset. 

## 4. Discussion

The current study presents a survey addressing the quality of life and working conditions of plastic surgeons in Switzerland. There were three major findings. The first is that the majority of Swiss plastic surgeons experience significant stress and endure long working hours. Even those without symptoms of depression, chronic fatigue, or burnout report high workloads that negatively impact their quality of life. The second major finding is that female surgeons are disproportionately affected by symptoms of depression or burnout (38% of female participants reported experiencing burnout compared to 22% of male participants), a trend that could become increasingly problematic given the rising number of women entering the medical profession [16,17]. The third major finding concerns younger surgeons who are particularly vulnerable to stress during holidays and leisure time (89% of surgeons aged 18–29 and 69% of those aged 30–44 reported feeling stressed in their leisure time compared to 59% of those aged 45–59 and 33% of those aged 60+). Nevertheless, the Swiss healthcare system relies on a motivated and healthy younger generation to ensure its future sustainability. 

### 4.1. Theoretical and Empirical Contexts: Work-Related Stress, Burnout, and Quality of Life in Surgery

The quality of life and occupational well-being of surgeons has emerged as a critical topic in the healthcare literature, particularly as workforce sustainability and physician mental health are increasingly recognized as determinants of healthcare quality and patient safety. The concept of burnout, as defined by the World Health Organization (WHO), reflects a response to chronic workplace stress that remains unmanaged, manifesting as emotional exhaustion, depersonalization, and a diminished sense of professional accomplishment [20]. This framework is central to understanding the findings of our study, which aligns with the existing literature showing that burnout is prevalent among surgeons across specialties and geographies.

Meta-analyses estimate that up to 34% of surgeons experience significant burnout symptoms, with direct links to medical errors, reduced quality of care, and compromised mental health [21,22]. In plastic surgery specifically, studies from the United States report burnout prevalence around 30%, with contributing factors including extensive working hours, reconstructive workloads, administrative burden, and insufficient institutional support [23]. These patterns mirror those observed in our Swiss sample, where 29% of participants reported burnout, a figure significantly higher than the estimated 18% in the general Swiss population.

This literature also emphasizes the vulnerability of certain subgroups—particularly female and early-career surgeons—to mental health challenges and reduced quality of life. Gender disparities in work–life integration, leadership access, and household responsibilities continue to shape occupational experiences in surgery. Women are disproportionately affected by burnout and are more likely to face structural obstacles in career advancement and personal life balance [23,24,25,26,27]. Our findings corroborate this: 38% of female plastic surgeons reported burnout symptoms compared to 22% of men.

Moreover, early-career professionals—particularly residents and junior consultants—face increased risk of work-related stress and dissatisfaction. These findings are consistent with the sociological theory of career-stage vulnerability, which posits that younger workers are more exposed to occupational pressures while still forming their professional identities. Our data show that younger surgeons (18–29 years) reported the highest levels of stress during personal time (89%), followed by those aged 30–44 (69%).

The literature further suggests that long working hours remain one of the most consistent predictors of physician distress [28,29,30,31]. In our study, the average weekly workload of 57.75 h exceeded contractual expectations by nearly 9 h, compounded by an additional 8.13 h during free time. Structural demands, including frequent on-call duties and administrative obligations, have been shown to drive dissatisfaction and foster disengagement, especially in hospital-based settings [32,33].

Career satisfaction, while generally high among surgeons, is closely tied to workplace autonomy, collegial support, and the ability to balance personal and professional life [15,34]. In our study, plastic surgeons in private practice reported significantly higher satisfaction with both career and compensation compared to hospital-employed peers. These differences are echoed in the literature, emphasizing the protective effects of autonomy and income stability on physician well-being.

Taken together, these findings support a biopsychosocial model of surgical well-being, in which systemic, interpersonal, and individual-level factors intersect to influence mental health, career satisfaction, and quality of life. Theoretical perspectives on organizational justice, gender equity, and professional identity development further contextualize our results. As burnout and stress continue to affect surgical professionals globally, interventions must go beyond individual coping strategies to include institutional reforms, mentorship structures, and equitable workplace policies.

In light of this framework, our study contributes novel national data on Swiss plastic surgeons, a population previously under-represented in the literature. It also reinforces calls for targeted structural changes in surgical practice, including reduced workload, enhanced autonomy, improved compensation, and, especially, gender- and career-stage-sensitive support programs.

### 4.2. Work-Related Stress and Burnout

Burnout, as defined by the World Health Organization (WHO), is a syndrome caused by chronic workplace stress that remains unmanaged, characterized by exhaustion, mental detachment from work, and reduced professional efficacy [20]. A large meta-analysis involving 3581 participants revealed that 3% of surgeons suffer from severe burnout, while up to 34% experience significant burnout in at least one dimension (e.g., emotional exhaustion, depersonalization, or reduced personal accomplishment) [21]. Burnout has been linked to an increased risk of medical errors and decreased mental quality of life among surgeons [22]. Approximately 30% of U.S. plastic surgeons experience burnout, linked to factors such as long hours, frequent night shifts, and reconstructive-focused practices. Burnout is associated with higher risks of medical errors, reduced quality of life, and career dissatisfaction [32,33].

In the current survey, 29% of plastic surgeons reported experiencing burnout, a rate higher than that of the general Swiss population (18%) and similar to the findings of Pulcrano et al., who examined the quality of life and burnout rates across various surgical specialties in a systematic review [2]. Younger surgeons, particularly residents and junior physicians, face a higher risk of burnout, depression, and chronic fatigue compared to those in senior positions. Early-career surgeons are also more likely to report poor quality of life [28,29].

Preventive measures, such as structured mentoring programs, increased control over working hours, and improved access to mental health resources, have been suggested to mitigate burnout [29]. Mentorship is vital in plastic surgery, particularly for fostering women’s leadership. Studies show that mentorship increases career satisfaction and academic output, while fellowships in subspecialties like microsurgery or aesthetics enhance marketability and career advancement [30]. For female physicians, targeted interventions include reducing gender bias, improving work–life integration, and providing mentorship, family leave, and childcare support [31]. There is strong evidence that burnout negatively affects patient care, organizational sustainability, and physician retention. Health organizations must prioritize evidence-based strategies, especially for residents and young physicians, to curb burnout and promote a healthier workforce [35,36]. Mentorship programs, such as the one recently introduced by the University of St. Gallen in collaboration with Swiss hospitals, aim to support young female residents in pursuing leadership and academic roles, further addressing these challenges [37]. 

Burnout remains a central issue in plastic surgery globally. Prendergast et al. reported that 45% of surgeons experience burnout, characterized by emotional exhaustion, depersonalization, and a reduced sense of personal accomplishment [10]. Similarly, Karanjkar et al. found that burnout among Indian plastic surgeons, although lower than global averages, is primarily associated with overwork and unrealistic expectations [38]. These findings align with the Swiss experience, where long working hours, administrative burdens, and complex patient expectations emerged as significant stressors. Addressing burnout requires systemic and individual interventions. Promoting work–life balance, providing mental health resources, and fostering a supportive workplace culture could mitigate some of these pressures. Regular wellness programs and peer-support networks have proven effective in reducing burnout in other healthcare settings [10,38]. 

### 4.3. Quality of Life and Physical Health Risks

Quality-of-life aspects, such as work, family, and social life, vary significantly depending on the age and professional role of plastic surgeons (e.g., residents, consultants, leading positions, or chief positions/practice owners). The current study highlights that younger female surgeons, particularly residents and junior physicians, are disproportionately affected by stressful working conditions, resulting in a noticeably reduced quality of life. 

This finding may be put into review by the findings of Balch et al., who emphasized the critical role of surgeons in exemplifying good health: “As surgeons, it is our responsibility to model good health for both our patients and the next generation of surgeons.” They elaborate: “To deliver the highest quality care to our patients, we must remain attentive, engaged in our work, and prepared to meet their needs” [39]. To achieve this, individuals, societies, and organizations must adopt and implement strategies that support work–life integration, ensuring the well-being of surgeons while maintaining high standards of patient care [24].

### 4.4. Gender Disparities and Work–Life Balance

Gender plays a complex role in work–life integration, influencing various aspects of professional and personal life [24]. In the context of academic surgery, women face distinct challenges, including access to collaborations and support networks, balancing family responsibilities with work, and navigating evolving perceptions of their roles. To foster equitable opportunities, it is essential to better understand the experiences of women in academic surgery and address barriers encountered by both genders [23]. While women report slightly lower career satisfaction compared to men (77% versus 82%) [24], previous studies have highlighted similar trends, suggesting that female surgeons are less likely to feel satisfied with their careers [27]. Interestingly, the findings of this study align with those trends, showing comparable satisfaction levels.

Research by Baptiste et al. revealed additional disparities: female surgeons are more likely than male to be married to a full-time professional (90% versus 37% among specialists; 82% versus 41% among residents) and are less likely to hold permanent positions. Furthermore, women disproportionately manage household responsibilities, such as childcare, meal preparation, grocery shopping, and holiday planning, while tasks like financial management and paying bills tend to be shared equally [25]. Addressing these gendered imbalances should be a priority, aiming to promote greater equity both at work and at home.

Ibrahim et al. highlighted the importance of work–life balance and mentorship, particularly for female medical students and surgeons [14]. Our findings corroborate these observations, with female surgeons expressing greater concerns about gender equity and family planning. Proactive policies to promote gender equity, such as flexible working arrangements and mentorship programs, are crucial to retaining diverse talent in the field.

While the representation of women in plastic surgery has grown from 4% in 1998 to approximately 22–28% in 2011, challenges persist, including delayed childbearing, higher divorce rates, and under-representation in leadership and academic authorship [26].

### 4.5. Working Hours

Our national survey also included questions about working hours. Previous studies have shown that time spent at the workplace strongly influences medical mistakes, depression, and burnout rates [40,41]. Tibble et al. found that surgeons are more than twice as likely to receive complaints compared to their physician peers, likely due to their involvement in complex surgical procedures and treatments [42]. Stressful, overbooked days and limited time for patient care outside the operating room may contribute to this trend. These factors were reflected in the current study, where participants reported working an average of 57.75 h per week, exceeding their contractual 48.87 h. Additionally, surgeons worked an average of 8.13 extra hours per week during their free time. Notably, 42% of participants expressed a desire to reduce their working hours, emphasizing the need for structural changes to improve work–life balance.

### 4.6. Career Satisfaction

Career satisfaction among plastic surgeons is deeply influenced by their professional environment. A U.S. survey found that only 4% of plastic surgeons regret their career choice, whereas in our study, this number is notably higher at 12%. Group practice settings and a balanced mix of cosmetic and reconstructive work significantly correlate with higher satisfaction [34]. Lin et al. described the challenges academic plastic surgeons face, including the undervaluation of teaching roles and increasing demands for clinical productivity [43]. Similarly, Sun et al. emphasized the lack of institutional support for educational activities, which are essential for fostering the next generation of surgeons [15]. Enhancing recognition and compensation for teaching efforts could improve job satisfaction and retention rates among Swiss academic surgeons.

Furthermore, conflicts in work–life integration contribute to career dissatisfaction, while collegial support for such efforts strongly correlates with higher career satisfaction [44]. Strengthening collegial support within medical teams is, therefore, essential. The current study reflects this, as 93.4% of respondents reported feeling supported in their professional environment, although the source of support (e.g., colleagues, family, or friends) was not specifically defined. This support likely plays a crucial role in career satisfaction, as the average job satisfaction among participants was 3.66 out of 5. However, 17.35% of respondents still indicated a lack of support, suggesting room for improvement.

Additionally, 63% of participants reported experiencing stress during their leisure time or holidays, with significant differences based on professional roles: 77.4% of residents, 73.9% of consultants, 81.8% of leading positions, and 42.9% of chief positions or practice owners reported feeling stressed. These findings are consistent with those of Bohrer et al., who argue for concerted efforts by hospitals, professional societies, and insurers to improve surgeons’ working conditions [41]. Additionally, measures such as preserving medical values, fostering a positive work climate, and providing proper training could significantly enhance surgeons’ quality of life [41].

**Figure 7:**
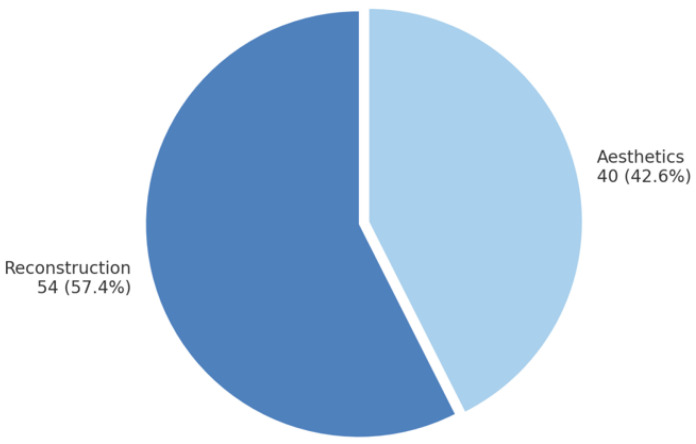
Areas of interest in plastic surgery.

The decision between a hospital career and private practice represents a major turning point for many plastic surgeons. Figure 7 illustrates the primary areas of interest within plastic surgery, as reported by the 94 survey participants who answered this optional question. The percentage values have been normalized to sum to 100% to reflect the true distribution of responses.

Our data show that plastic surgeons in private practice are significantly more satisfied with their professional situation (average job satisfaction: 4.0 vs. 3.52 in hospitals, *p* = 0.017). This could be particularly linked to better pay (salary satisfaction: 3.82 in private practice vs. 2.86 in hospitals). Interestingly, 40% of hospital-based surgeons and 40% of private practitioners stated that they would be even more satisfied with greater autonomy, indicating that decision-making freedom remains an important factor for both groups. The statistically significant difference in job satisfaction between hospital and private practice (*p* = 0.017) supports the idea that private practice tends to be more satisfying, likely due to factors such as the salary and autonomy.

Career satisfaction remains high overall, as only 12% of respondents stated they would no longer choose plastic surgery as a profession, a rate comparable to findings in the literature for general surgeons (21% of female and 13% of male general surgeons) [27]. Such numbers underscore the need for targeted interventions to reduce stress and improve work–life integration, ensuring sustainable career satisfaction across all levels of seniority.

### 4.7. Comparison of Plastic Surgery Versus Hand Surgery

Since 2015, the specializations in plastic surgery and hand surgery have been separated in Switzerland, allowing physicians to obtain board certification in either discipline independently. Given that both fields originally emerged from a common training path, it is particularly interesting to compare them, as the conditions were still identical less than a decade ago. The quality of life and working conditions of surgeons have been a topic of growing importance, as highlighted in a recent national survey of Swiss hand surgeons, which provided valuable insights into the challenges faced by professionals in this field [1]. A comparison between plastic and hand surgery revealed notable similarities and differences in working conditions, stress levels, and career satisfaction. In terms of working hours, plastic surgeons reported an average of 48.87 contractual hours per week but worked 57.75 h on average, with an additional 8.13 h spent on work-related tasks during their free time. Similarly, hand surgeons experienced highly variable weekly working hours ranging from 30 to 82 (average 55 h), with additional hours during leisure time ranging from 0 to 30 (average 6.6 h). 

These demanding working hours contribute to high levels of stress and burnout in both specialties. Burnout rates were comparable, with 29% of plastic surgeons and 29.1% of hand surgeons reporting experiences of burnout, chronic exhaustion, or depression. Stress during leisure time was significant in both groups; 63% of plastic surgeons felt stressed during their holidays, while for hand surgeons, stress was highest among residents (84%), followed by senior physicians (50%), leading physicians (27.6%), and senior consultants/practice owners (40.6%). Career satisfaction showed slightly different trends. Among plastic surgeons, the average career satisfaction score was 3.66 on a scale from 1 to 5, and 42% expressed a desire for fewer working hours. For hand surgeons, 89.1% stated they would choose the specialty again, and better pay (98.2%) and reduced on-call duties (56.4%) were identified as the top two factors to improve job attractiveness. The impact of work on personal life was another common concern. In plastic surgery, 63% reported negative effects on private relationships compared to 85.4% of hand surgeons. Despite these challenges, 93.4% of plastic surgeons and 90% of hand surgeons felt supported by their professional and personal environments. Both specialties highlighted similar priorities to improve work conditions: better compensation, reduced on-call responsibilities, and greater autonomy. However, plastic surgeons emphasized autonomy and fewer time-tracking regulations, whereas hand surgeons prioritized fewer on-call duties and better pay. 

In conclusion, while both fields face comparable challenges, stress appears to be more prevalent in hand surgery, particularly among residents. Addressing these issues through reduced workloads, improved compensation, and targeted support programs could significantly enhance career satisfaction and work–life integration for both specialties. Achieving this balance is essential not only for surgeons’ well-being but also for maintaining high-quality patient care [1].

### 4.8. Strengths and Limitations

The present study’s strength lies in its large sample size of plastic surgeons across Switzerland (*n* = 3) and its response rate of 22.8%, which compares favorably with similar studies [45]. It offers valuable new insights into factors affecting the quality of life for plastic surgeons, an area that has been largely underexplored in prior research. Nevertheless, as with most survey-based research, recall bias may have influenced the participants’ responses. Moreover, the questionnaire used in this study was not statistically validated, and some questions were open to interpretation (e.g., the exact meaning of “social life”). To enhance future research, it will be important to refine survey designs, provide clearer definitions, and validate the instruments rigorously to strengthen the reliability and applicability of the findings.

### 4.9. Recommendations and Future Directions

Promoting mental health and preventing burnout are vital for the well-being of plastic surgeons, although we think this is true for all employees in the medical field. Structured wellness programs, peer support, and a culture prioritizing mental health and work–life balance can mitigate professional strain. Ergonomics and physical health can be improved through investments in ergonomic tools, training, and intraoperative microbreaks to prevent injuries. Career satisfaction can be enhanced by compensating educational and mentorship roles and reducing bureaucratic burdens that detract from clinical and teaching responsibilities. Advancing gender equity requires flexible work options and mentorship programs to support female surgeons and foster leadership opportunities. Expanding access to care involves incentivizing rural practices and leveraging telemedicine to address healthcare disparities, ensuring underserved populations receive timely and effective treatment. Therefore, the findings of this survey could help guide the development of future targeted interventions to improve quality of life and working conditions for plastic surgeons. Ultimately, improving the working conditions of doctors not only benefits the practitioners themselves but also ensures better care for the patients they serve [46,47].

## 5. Conclusions

The quality of life of plastic surgeons in Switzerland is heavily impacted by demanding workloads and long working hours. Our study showed that residents, consultants, and female surgeons are particularly vulnerable, reporting higher rates of depression, burnout, and chronic fatigue compared to those in leadership roles, senior consultants, practice owners, and male surgeons. Improved pay and reduced on-call duties were highlighted as ways to make acute care hospital work more attractive. Despite these challenges, 87.8% of respondents would choose plastic surgery as their medical specialization again. Addressing burnout, enhancing career satisfaction, and improving work environments are essential to supporting practitioners and to ensure high-quality patient care. Future research should examine the long-term effects of targeted interventions on surgeon well-being and job satisfaction.

## Figures and Tables

**Table 1:** Characteristics of participants.

Age	
18–29	10 (9.8%)
30–44	39 (38.2%)
45–60	22 (21.6%)
60+	4 (3.9%)
**Gender**	
Male	60 (58.8%)
Female	42 (41.2%)
**Relationship**	
Partnership	76 (74.5%)
Single	17 (16.7%)
Divorced	6 (5.9%)
**Children**	
Yes	46 (45.1%)
No	56 (54.9%)

**Table 2:** Work-related participants’ characteristics.

Position	
Resident	32 (31.4%)
Consultant	23 (22.6%)
Leading position	11 (10.8%)
Chief position	6 (5.9%)
Activity in private practice	28 (27.5%)
**Hospital/practice categories**	
Category A	47 (46.1%)
Category B	16 (15.7%)
Category C	5 (4.9%)
Practice	30 (29.4%)

**Table 3:** Participants’ satisfaction.

**Satisfaction in terms of social life**	
Not satisfied at all	6 (5.9%)
Insufficient	16 (15.7%)
Moderate	42 (41.2%)
Good	30 (29.4%)
Completely satisfied	6 (5.9%)
**Satisfaction in terms of family life**	
Not satisfied at all	0 (0%)
Insufficient	14 (13.7%)
Moderate	29 (28.4%)
Good	43 (42.2%)
Completely satisfied	12 (11.8%)
**Satisfaction in terms of job/career**	
Not satisfied at all	1 (1%)
Insufficient	11 (10.8%)
Moderate	25 (24.5%)
Good	48 (47.1%)
Completely satisfied	16 (15.7%)
**Satisfaction in terms of salary**	
Not satisfied at all	8 (7.8%)
Insufficient	27 (26.5%)
Moderate	23 (22.6%)
Good	27 (26.5%)
Completely satisfied	15 (14.7%)

## Data Availability

The data that support the findings of this study are not publicly available due to privacy and confidentiality considerations. However, de-identified data may be made available upon reasonable request to the corresponding author, subject to institutional and ethical approval.
